# Direct Current Coupled Recordings of Cortical Spreading Depression Using Silicone Probes

**DOI:** 10.3389/fncel.2017.00408

**Published:** 2017-12-21

**Authors:** Azat Nasretdinov, Nailya Lotfullina, Daria Vinokurova, Julia Lebedeva, Gulshat Burkhanova, Kseniya Chernova, Andrey Zakharov, Roustem Khazipov

**Affiliations:** ^1^Laboratory of Neurobiology, Department of Human and Animal Physiology, Kazan Federal University, Kazan, Russia; ^2^Institut de Neurobiologie de la Méditerranée (INMED)—INSERM, UMR901, Aix-Marseille University, Marseille, France

**Keywords:** electroencephalography, DC recordings, silicone probes, spreading depression, epilepsy, migraine, traumatic brain injury, brain ischemia

## Abstract

Electrophysiological assessment of infraslow (<0.1 Hz) brain activities such as cortical spreading depression (SD), which occurs in a number of pathologies including migraine, epilepsy, traumatic brain injury (TBI) and brain ischemia requires direct current (DC) coupled recordings of local field potentials (LFPs). Here, we describe how DC-coupled recordings can be performed using high-density iridium electrode arrays (silicone probes). We found that the DC voltage offset of the silicone probe is large and often exceeds the amplifier input range. Introduction of an offset compensation chain at the signal ground efficiently minimized the DC offsets. Silicone probe DC-coupled recordings across layers of the rat visual and barrel cortices revealed that epipial application of KCl, dura incision or pinprick TBI induced SD which preferentially propagated through the supragranular layers and further spread to the granular and infragranular layers attaining maximal amplitudes of ~−30 mV in the infragranular layers. SD at the superficial cortical layers was nearly two-fold longer than at the deep cortical layers. Continuous epipial KCl evoked multiple recurrent SDs which always started in the supragranular layers but often failed to propagate through the deeper cortical layers. Intracortical KCl injection into the infragranular layers evoked SD which also started in the supragranular layers and spread to the granular and infragranular layers, further indicating that the supragranular layers are particularly prone to SD. Thus, DC-coupled recordings with silicone probes after offset compensation can be successfully used to explore the spatial—temporal dynamics of SD and other slow brain activities.

## Introduction

Electrical brain activity is characterized by signals occurring in a wide range of frequencies including slow and infraslow (<0.1 Hz) frequencies (Buzsáki and Draguhn, 2004). Thus, the phenomena of cortical spreading depression (SD) or slow activity transients (SATs) develop on the time scale of tens of seconds, while negative local field potential (LFP) shifts during ischemic depolarization in the necrosis zones may persist even longer (Somjen, 2001; Vanhatalo et al., 2005a; Colonnese and Khazipov, 2010; Ayata and Lauritzen, 2015; Dreier and Reiffurth, 2015; Dreier et al., 2017; Hartings et al., 2017a). These slow brain activities are significantly compromised during high-pass filtering using conventional >0.5 Hz electroencephalography and their neurophysiological assessment requires direct current (DC)-coupled recordings including stable, low-noise non-polarizing electrodes and DC amplifiers (Tallgren et al., 2005; Vanhatalo et al., 2005b; Dreier et al., 2017; Hartings et al., 2017a). AgCl/Ag electrodes are considered the optimal electrodes for DC-coupled electrographic recordings and AgCl multielectrode arrays (such as 16-channel Prohaska’s ottosensors) or multiple glass pipette microelectrodes positioned at different depth have been successfully used to characterize the spread and current-source density (CSD) of the DC shifts during seizures and SD in the hippocampus (Wadman et al., 1992; Herreras and Somjen, 1993a; Herreras et al., 1994; Makarova et al., 2008). Other materials (such as gold, platinum, or steel) are characterized by large voltage offsets, high noise and polarization and therefore are thought to be poorly suitable for DC-coupled recordings (Tallgren et al., 2005). These observations question the suitability of high-density silicone multichannel arrays which typically use platinum or iridium electrodes for DC-coupled recordings.

SD is a pathological wave of collective neuronal depolarization that slowly propagates through the gray matter of the cerebral cortex. SD is associated with a massive breakdown of ionic gradients and nearly complete loss of the membrane potential so that the neurons recruited by SD rapidly go into the state of depolarization block (Herreras and Somjen, 1993b; Somjen, 2001; Canals et al., 2005; Dreier, 2011; Pietrobon and Moskowitz, 2014; Ayata and Lauritzen, 2015). Large, up to tens of millivolts in amplitude and transient, ~1 min in duration, negative LFP shifts are the hallmark of SD. SD occurs during and directly participates in a number of pathologies including migraine, traumatic brain injury (TBI), brain ischemia and epilepsy (Wadman et al., 1992; Bragin et al., 1997; Lauritzen et al., 2011; Dreier and Reiffurth, 2015; Hartings et al., 2017b). SD involves several synaptic and non-synaptic mechanisms with elevation of extracellular potassium playing instrumental roles in SD initiation and propagation. Application of high KCl on the cortical surface readily induces SD which propagates horizontally through the gray matter from the site of KCl application. In the hippocampus, SD displays remarkable layer-specific features with the maximal negative LFP shifts and associated sinks observed in the layers containing apical and basal dendrites of CA1 pyramidal cells (Wadman et al., 1992; Makarova et al., 2008). However, how SD spreads vertically and whether SD features differ between the layers in the neocortex remains less well understood. In Leão (1944, 1947) initial descriptions, neocortical SD was more easily induced and propagated faster in the superficial cortical layers that was supported by further studies using brain slices *in vitro* (Basarsky et al., 1998; Joshi and Andrew, 2001; Kaufmann et al., 2017). Simultaneous recordings at different cortical depths *in vivo* supported preferential propagation of SD either in deep (Richter and Lehmenkühler, 1993) or in superficial (Kaufmann et al., 2017) cortical layers, a conclusion based on different frequency of recurrent SDs at different cortical depths. However, electrode arrays in these previous studies have not been vertically aligned which precludes the determination of SD onsets at different depths within the same cortical column.

In the present study, we attempted to address SD properties within a cortical column using linear multichannel silicone probes. However, we faced the problem of large DC offsets of iridium electrodes which often exceeded the amplifier input range. Introduction of an offset compensation chain at the signal ground efficiently minimized these DC offsets. This enabled us to characterize the propagation of SD across layers of the cortical column, and show that SD propagates preferentially through the supragranular layers before invading the entire cortical column and that SD displays prominent layer-specific features.

## Materials and Methods

This work was carried out in accordance with EU Directive 2010/63/EU for animal experiments, and all animal-use protocols were approved by the French National Institute of Health and Medical Research (INSERM, protocol N007.08.01), and by Kazan Federal University on the use of laboratory animals (ethical approval by the Institutional Animal Care and Use Committee of Kazan State Medical University N9-2013). Wistar rats from postnatal days P20–60 were used in electrophysiological experiments. Preparation of the animals for head-restrained recordings and the recording setup were as described previously (Khazipov et al., 2004; Minlebaev et al., 2011; Akhmetshina et al., 2016) with some modifications. In brief, under isoflurane anesthesia (5% induction, 1.5%–2% maintenance) the skin above the skull was removed, the periosteum removed using a scalpel and Hemostab, and the skull was covered with dental cement (Grip Cement, Caulk Dentsply, DE, USA), leaving a 3 × 7 mm rostro-caudal window above the left cortex. Then isoflurane anesthesia was discontinued and the animals were administered urethane (1.5 g/kg, i.p.) and warmed. A metal ring was fixed to the skull by dental cement and via ball-joint to a magnetic stand. Animals were heated via a thermal pad (35–37°C). A chloride silver wire placed in the cerebellum or temporal muscles served as a ground electrode. Two cranial windows were drilled in the skull, one above the visual cortex (VCx) for recordings with a silicone probe at coordinates AP –4.5 mm and L 4 mm (Paxinos and Watson, 2007; Khazipov et al., 2015), and another window was made 2–3 mm more rostral or more caudal from the recording window (at an average distance of 2.6 ± 0.4 mm; *n* = 9), through which SD was induced by epipial application of 0.05–1 M KCl solution. For recordings from the barrel cortex (BCx), a cranial window was drilled at coordinates AP –2.5 mm and L 5.5 mm and a 1 M KCl drop was applied epipially through the second window 3–5 mm more caudal from the recording window. In most of the experiments KCl was applied briefly and as soon as SD was observed it was rinsed three times with PBS. In a subset of animals 1 M KCl was applied continuously for 2 h. In another group of four animals 1 M KCl was injected intracortically at a depth of 1400–1500 μm using a Micro4 microsyringe pump (WPI, Sarasota, FL, USA) at 250–500 nl/min for 1–2 min. Also, SD was recorded in response to dura incision during preparation of the remote window, and pinpricks by inserting a broken glass pipette with a 30 μm tip or 100 μm needle to a depth of 500 μm.

Recordings of the LFP were performed using linear multichannel arrays (silicone probes, 413 μm^2^ iridium electrode surface, 100 μm electrode separation distance, Neuronexus Technologies, Ann Arbor, MI, USA). The signals were amplified and filtered at 0 Hz–9 kHz using a DigitalLynx (Neuralynx, Bozeman, MT, USA) amplifier set in DC mode (input range ±131 mV), digitized at 32 kHz and saved on a PC for *post hoc* analysis using custom-written functions in Matlab (MathWorks, Natick, MA, USA). In the experiments in a dish, voltage was also measured at a distance of 100 μm from one channel of the silicone probe using glass pipette/AgCl/Ag electrodes connected to the patch-clamp amplifier. Glass pipette electrodes were pulled from borosilicate glass capillaries (GC150F-15, Clark Electromedical Instruments) and had a resistance of 2–3 MΩ when filled with ACSF of the following composition: (in mM): NaCl 126, KCl 3.5, CaCl_2_ 2, MgCl_2_ 1.3, NaHCO_3_ 25, NaH_2_PO_4_ 1.2 and glucose 11. Electrodes were connected via chlorided silver wire to the headstage of a MultiClamp700B patch-clamp amplifier (Axon Instruments, Union City, CA, USA). Recordings were performed in current-clamp mode, filtered at 0–3 kHz and digitized using Digidata1440 (Axon Instr., Union City, CA, USA).

Calculation of SD parameters was performed as follows: the original signal at each channel was downsampled to 1 kHz and linear trends were removed using a 300 s long sliding window with 10 s overlap. Resulting signals were smoothed by the 1000-point moving average filter and the first LFP derivatives were calculated. For each recording site, the local negative peak time of the first LFP derivative within the 20 s time window preceding the negative SD peak was considered as SD onset. Velocity of vertical SD propagation was calculated from onset values as distance between neighboring recording sites (100 μm) divided by the SD onset delays between the corresponding channels. Baseline level was calculated for each recording site as the mean LFP value in −20 s to −10 s time window preceding SD onset. SD amplitude was calculated as the maximal negative LFP peak from the baseline. Duration of SD was calculated as the duration during which SD was 50% of it’s maximum amplitude. SD surface was defined as an area bounded by 50% of SD amplitude level. SD gradient was calculated as the value of the first LFP derivative value at the SD onset time, normalized to SD amplitude. SD afterhyperpolarization (AHP) was detected in a 2 min long time window after SD onset and its amplitude was calculated relative to the baseline. CSD analysis across the depth was then performed on the averaged signals to eliminate volume conductance and localize synaptic currents. It was computed for each recording site according to a differential scheme for second derivative and smoothed with a triangular kernel of length 4 (Freeman and Nicholson, 1975).

## Results

The aim of the present study was two-fold: first, to verify the suitability of silicone probes for DC-coupled recordings and second, to use linear multichannel silicone probes to characterize vertical propagation and layer-specific features of SD in the visual and barrel cortices.

**Figure 1:**
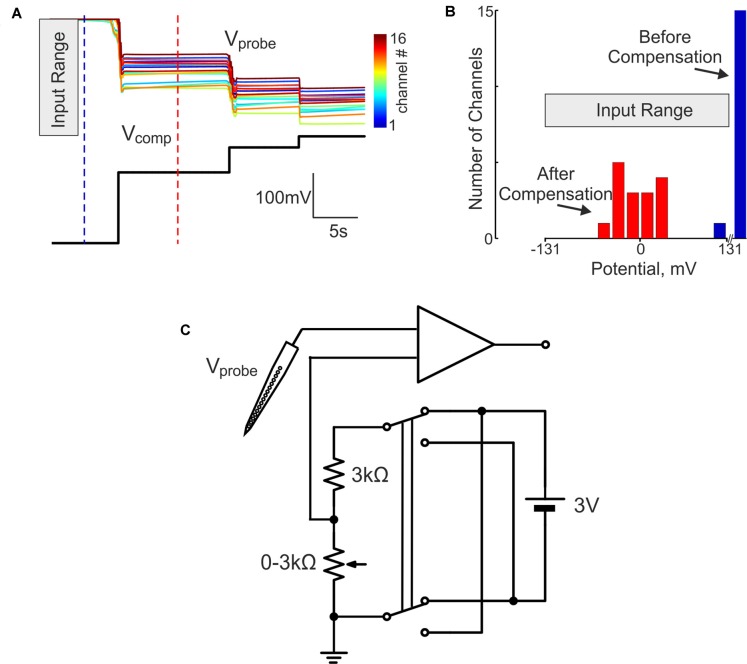
Direct current (DC) silicone probe offset compensation in the dish. **(A)** Top, color-coded voltage traces from 16 channels of the silicon probe (V_sil.probe_) placed in saline at different holding levels of compensatory potential (V_comp_, black trace below). **(B)** Histogram of the voltage values at the silicone probe in saline before (blue) and after (red) the offset compensation. **(C)** Circuit diagram for the DC-offset compensation. Compensatory potential is applied to the point between the ground electrode and the ground input of the amplifier and its size is manually controlled by the potentiometer within a compensation range of 0 ± 1.5 V.

**Figure 2:**
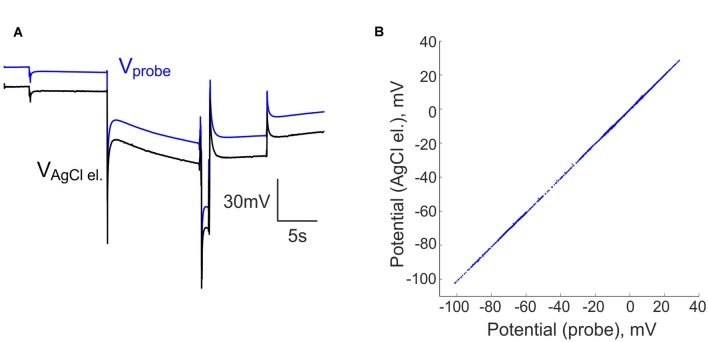
Comparison of the voltage recordings using silicone probe and AgCl-electrode. **(A)** Voltage traces recorded simultaneously by an ACSF-filled glass-pipette/AgCl electrode (black trace) and a silicone probe (blue trace) in saline at different levels of compensatory potential with a common ground electrode. **(B)** Voltage signals recorded by an AgCl/Ag electrode vs. a silicone probe in saline at different levels of compensatory potential. Voltage at the silicone probe was corrected for baseline offset.

In our initial attempts to record brain activity with silicone probes in a DC-coupled manner, we faced the problem of large DC offsets that often exceeded the amplifier range (±131 mV). The problem of variable and saturating DC offsets also persisted when the silicone probes were placed in saline in a dish (Figures 1A,B). In addition to the common offset, voltage values also differed between the sites of silicone probe with a range of ±20 mV. To compensate for the saturating offsets, we introduced a compensation chain between the combined reference/ground electrode and the ground input of the amplifier (Figure 1C). The offset compensation chain was powered by two 1.5 V alkaline AA type batteries and manually controlled by a 3 kOhm potentiometer. Example traces of the voltage values at 16 channels of the silicone probe before and after voltage offset compensation are shown in Figure 1A. Although the voltage values varied between the channels after the common voltage offset compensation, they all shifted into the recording range of ±131 mV (Figure 1B). After the baseline offset correction, voltage signals recorded by the silicone probe matched those recorded with the ACSF-filled glass-pipette/AgCl/Ag electrodes (Figure 2).

**Figure 3:**
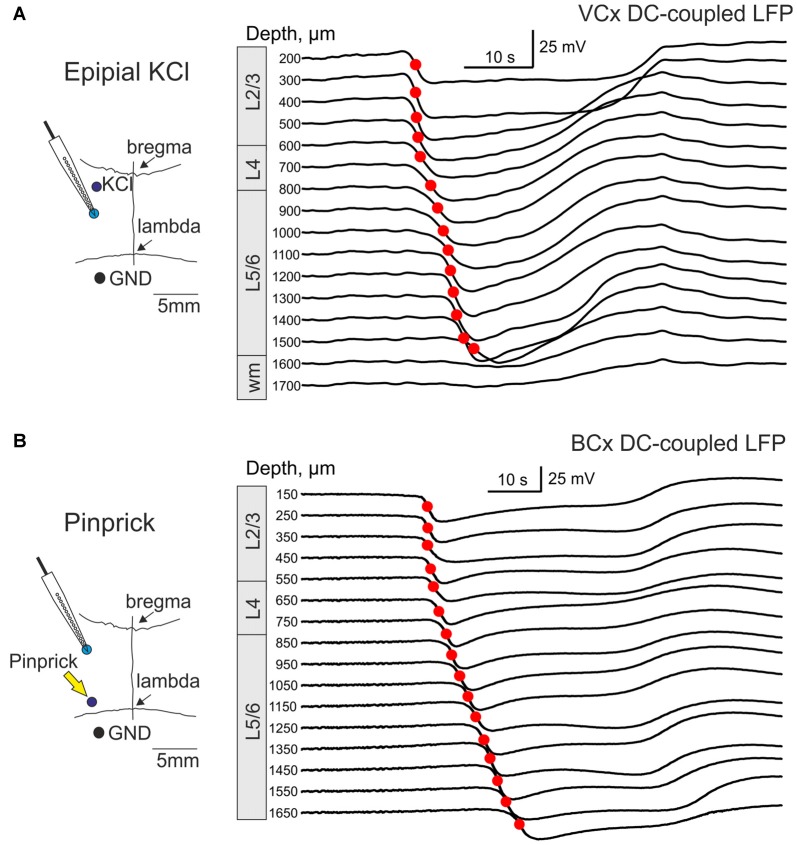
Vertical propagation of spreading depression (SD) in the visual and barrel cortex (BCx). **(A)** Left, Scheme of the experiment. A 16-channel silicone probe is vertically inserted into visual cortex (VCx). SD is induced by epipial application of 1 M KCl 3.5 mm anterior to the recording site. The ground electrode is placed in cerebellum. Right, Example traces of KCl-induced SD at different depths from the cortical surface in the VCx (cortical layers are indicated on the left) recorded by a 16-channel linear silicone probe (100 μm vertical separation distance). Red circles indicate maximal slope of the raising phase of SD at each trace, which were used for SD onset detection. **(B)** Scheme of SD recordings from the cortical column in the BCx (left). SD was induced by pinprick 3–5 mm caudal from the recording site. Right, Pinprick-induced SD at different depths from the cortical surface in the BCx.

**Figure 4:**
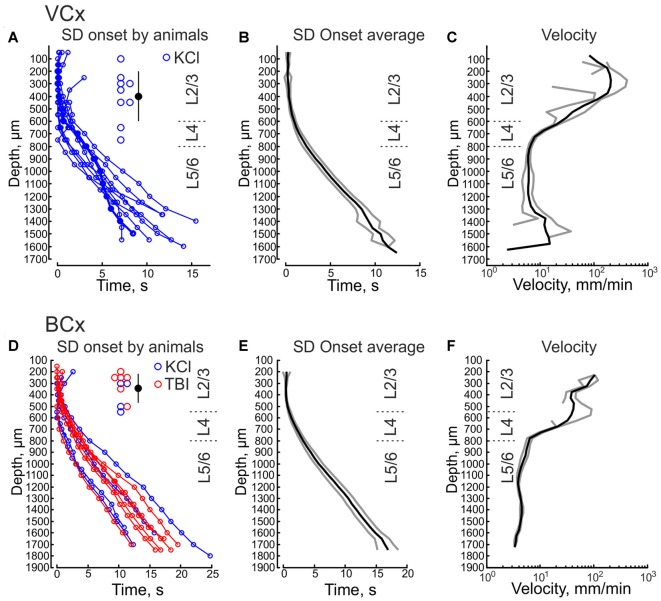
Onset of SD at different depths in the visual and barrel cortices. **(A,B)** Epipial KCl-induced SD onset as a function of cortical depth in the VCx. Pooled data from nine animals **(A)** and group average (**B**, mean ± SE). Time = 0 corresponds to the earliest SD onset detected through all channels. **(C)** Average velocity of vertical SD propagation calculated from onset values as a function of cortical depth in the VCx (mean ± SE, *n* = 9). **(D,E)** Epipial KCl (blue) and traumatic brain injury (TBI; pinprick and dura incision, red) induced SD onset as a function of cortical depth in the BCx. Pooled data from 10 animals **(D)** and group average (**E**, mean ± SE). **(F)** Average velocity of vertical SD propagation calculated from onset values as a function of cortical depth in the BCx (mean SE, *n* = 10). Insets on **(A,D)** show the depth values of the channels where the earliest SD onset was detected and corresponding mean depth value for the earliest SD onset ± SD.

**Figure 5:**
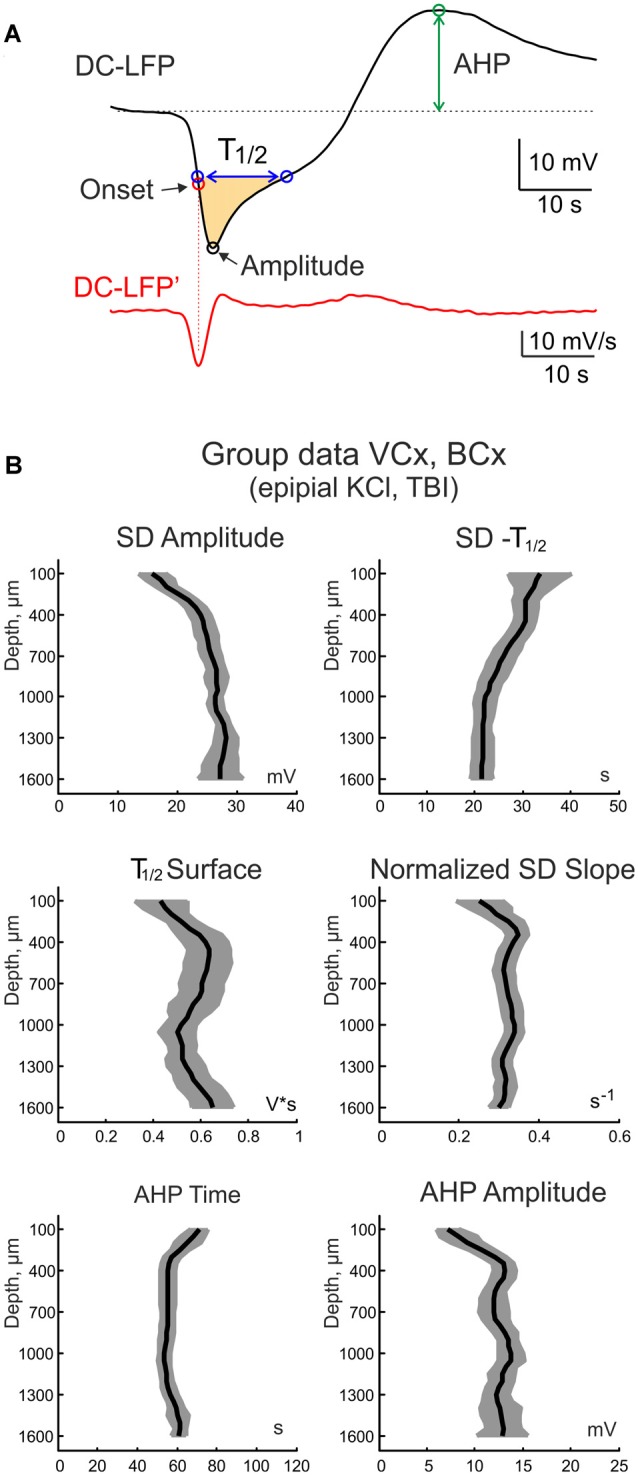
Parameters of SD at different cortical depth. **(A)** Example trace of SD (top black trace) and its first derivative (bottom red trace). The horizontal dashed line shows the baseline. The following SD parameters were calculated: SD amplitude, SD half-duration (SD—T_1/2_), surface of the half-negative SD phase (T1/2 surface, yellow-colored; SD slope; afterhyperpolarization (AHP) time from the shortest SD onset and AHP amplitude. **(B)** Group data on SD parameters at different cortical depths (mean ± SE; *n* = 19 rats; pooled data from epipial KCl and TBI induced SD in the visual and barrel cortices).

We further performed DC-coupled recordings of high-potassium induced SD using silicone probes in the cortex of urethane-anesthetized rats *in vivo*. Sixteen-channel linear silicone probes with 100 μm separation distance between the channels were inserted into the VCx or BCx to record extracellular LFP through all cortical layers. SD was evoked by application of 0.05–1 M KCl solution at the cortical surface at a distance of 2–3.5 mm from the recording site (Figure 3A). SD waves were recorded in the VCx and BCx 88 ± 23 s and 76 ± 10 s after epipial KCl application, respectively (Figure 3A). Onset of SD was determined as the time when the maximal rate of the negative voltage deflection during SD was attained at each channel (marked by red circles in Figures 3A,B) that corresponds to the negative peak of the DC-LFPs’ first derivative (Figure 5A, red trace). In the experiment shown in Figure 3A, SD was first observed in the supragranular layers, and it spread to the granular and infragranular layers. SD with a similar vertical propagation pattern was also observed in response to dura incision or pinprick at the remote cranial window (*n* = 6; Figure 3B). To characterize the vertical SD delays within the cortical column the earliest SD onset was taken as *T* = 0 and SD onset values at each channel were presented as the delay of SD onset from the earliest SD onset. The resulting SD onset delays obtained at different cortical depths in VCx and BCx are shown in Figures 4A,B,D,E, respectively. In VCx, epipial KCl-induced SD onset was first typically observed in L2/3 (*n* = 7/9 rats) or in L4 (*n* = 2/9 rats), at an average depth of 400 ± 70 μm (Figure 4A, inset). In BCx, SD onset was also first observed in L2/3 (*n* = 9/10 rats) or in L4 (*n* = 1/10 rats), at an average depth of 345 ± 40 μm (Figure 4D, inset; pooled data on epipial KCl application, dura incision and pinprick-induced SD). SD further spread to the surface and to depth, invading the entire column and fading in the white matter of all animals. Velocity of the vertical SD propagation was highest (~50–200 mm/min) in the supragranular layers 2/3 and it slowed down to ~4–6 mm/min in the infragranular layers (Figures 4C,F; note semilogarithmic scale to make more visible all velocity values). While the speed of SD wave in the infragranular layers is close to the horizontal SD propagation speed values reported previously (Somjen, 2001; Dreier and Reiffurth, 2015; Guedes et al., 2017; Kaufmann et al., 2017) and likely reflects “true” vertical propagation from the supragranular to infragranular layers, unusually high values of the velocity of vertical SD propagation within the supragranular layers likely reflect the SD front, which horizontally moves, with little jitter, from the initiation site through the entire depth of the superficial layers.

The electrographic SD phenotype varied at different cortical depth. At the cortical surface SD typically had a U-shape with up to a tens of seconds long, step-like negative LFP shift. In the deep layers, it more resembled a V-wave with a relatively short negativity transient curtailed by AHP. With the aim of characterizing and comparing SD properties at different cortical depths, we calculated the following SD parameters (Figure 5A): SD amplitude (the peak negativity attained during SD), SD half-duration (SD—T_1/2_, SD duration at its half-amplitude), surface of the half-negative SD phase (T_1/2_ surface, yellow-colored in Figure 5A); SD slope (maximal rate of SD’s negative phase); AHP time (the time of AHP peak from the SD onset) and AHP amplitude. Averaged values of these parameters across cortical depth for the epipial KCl and TBI—induced SD in VCx and BCx are shown in Figure 5B. Among the most prominent depth-dependent SD parameters were the following: (i) the SD amplitude was characterized by a bell-shape depth profile with the maximal value of ~30 mV attained in the deep cortical layers that was almost twice the SD amplitude at the cortical surface; (ii) The SD half-duration progressively shortened from the cortical surface (~40 s) to depth (~20 s); and (iii) deep cortical layers displayed stronger AHP than the superficial layers.

**Figure 6:**
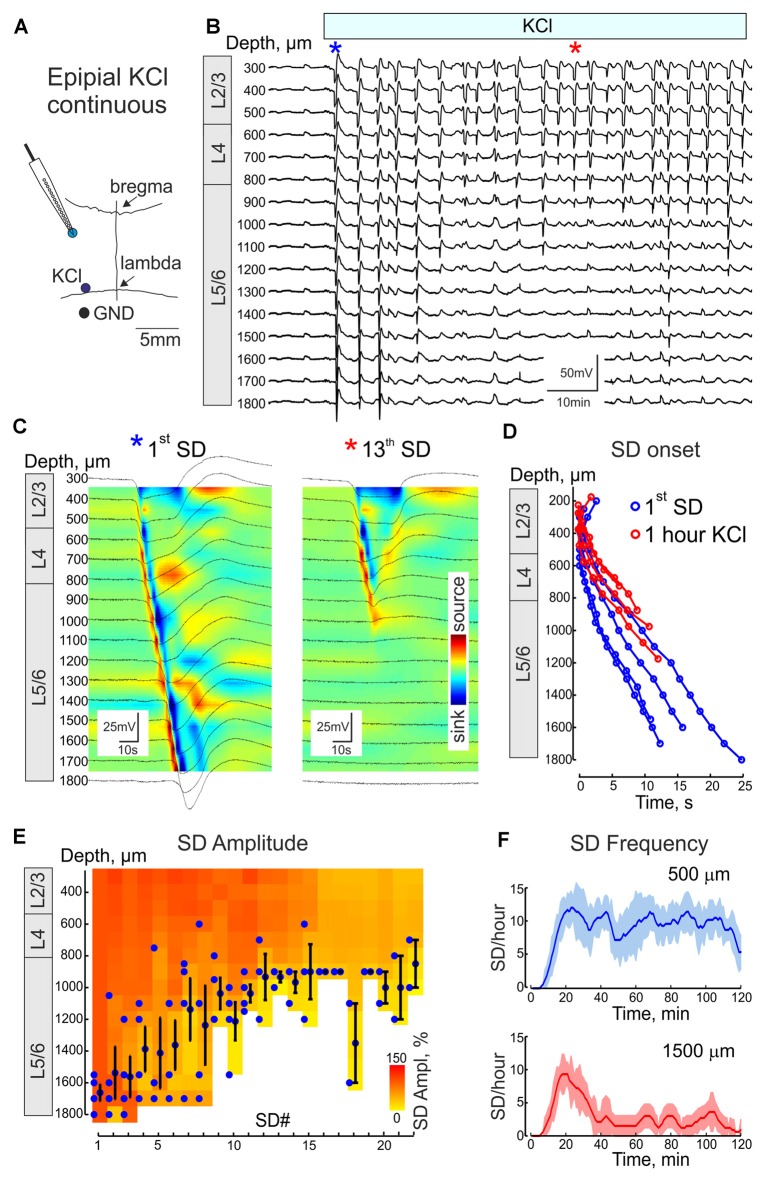
Vertical propagation of continuous epipial KCl-induced recurrent SDs in the BCx. **(A)** Scheme of SD recordings from the cortical column in the BCx. Recurrent SDs was induced by continuous 1 M KCl application 3–5 mm caudal from the recording site. **(B)** Example traces of SDs during 2 h of KCl application. The first and 13th SDs are marked by the blue and red asterisks and are shown on an expanded time scale on **(C)**. **(C)** The first and the 13th SDs at different depths from the cortical surface in the BCx overlaid on the color-coded current-source density (CSD) plot. **(D)** Plot of the first SD (blue) and SD 1 h after KCl application (red), onset times as a function of cortical depth (pooled data from four animals). **(E)** Vertical propagation of recurrent SDs as a function of the SD number. Blue circles indicate the deepest channel where SD was detected (each dot corresponds to an individual animal), Black circles and error bars indicate mean ± SE for each SD. Mean SD amplitude at each channel is color-coded. Note that recurrent SDs tend to recruit only the superficial cortical layers. **(F)** SD frequency as a function of time after the onset of KCl application at depth 500 μm and 1500 μm.

We further explored the features of recurrent SDs in BCx evoked by continuous remote epipial KCl application during 2 h (Figures 6A,B). Recurrent SDs occurred at a frequency of 9.0 ± 2.0 SDs per hour (*n* = 77 SDs from four rats; Figure 6F) and displayed remarkable changes in the propagation pattern through the time course of KCl application (Figures 6C,D). While several (from 1 to 8, mean 3.5 ± 1.5) initial SDs propagated through the entire cortical depth from the superficial to deep layers, the following SDs were mainly restricted to the supragranular and granular layers and their downward propagation was typically aborted at variable depth in the infragranular layers (on average, at 1120 ± 60 μm; *n* = 45 SDs from four rats, >60 min after the onset of continuous KCl application; Figures 6D,E). As a result of only occasional propagation of recurrent SDs from the superficial to deep layers, the rate of SD occurrence in the supragranular layers (9.0 ± 2.0 SDs/h) was nearly three-fold higher than in the infragranular layers (2.9 ± 0.6 SD/h; *n* = 36 SDs from four rats; Figure 6F). All recurrent SDs were initiated at the superficial layers through the entire time course of continuous KCl application (Figure 6D). The CSD profile of recurrent SDs, which was characterized by the most prominent frontal sink at the SD peak and second sink during SD rebound, was essentially unchanged in the superficial layers during aborted recurrent SDs (Figure 6C).

**Figure 7:**
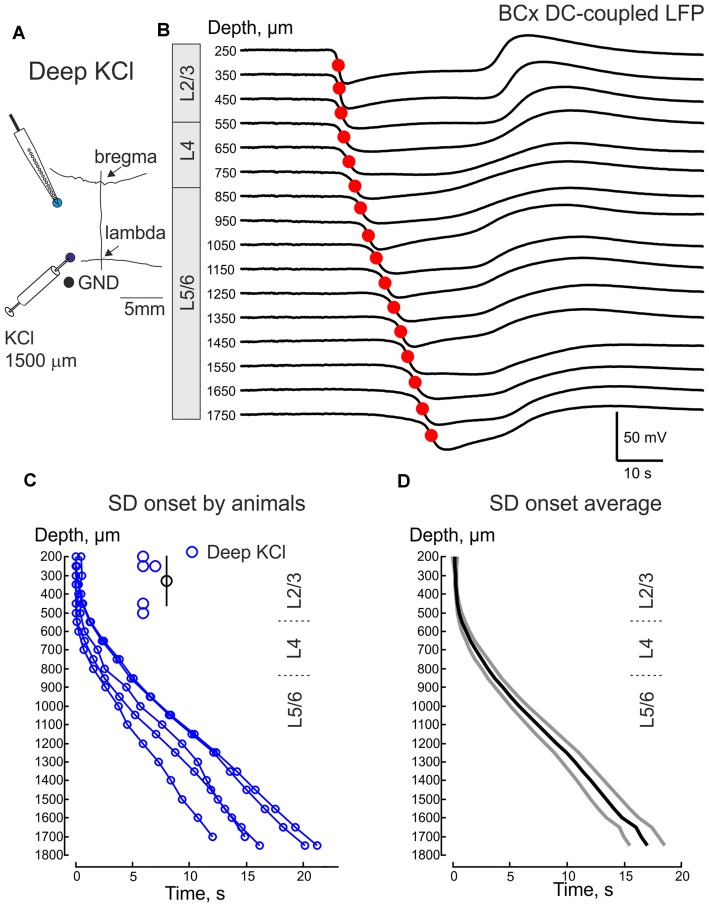
SDs in the BCx evoked by remote injection of KCl to the infragranular layers. **(A)** Scheme of the experiment. Recordings are performed from the BCx and SD is induced by injection of 1 M KCl at 1500 μm depth 3–5 mm caudal from the recording site. **(B)** Example traces of deep KCl-induced SD at different depths in BCx. **(C,D)** Deep KCl induced SD onset as a function of cortical depth in BCx. Pooled data from five animals **(C)** and group average (**D**, mean ± SE). Insets on **(A,D)** show the depth values of the channels where the earliest SD onset was detected and the corresponding mean depth value for the earliest SD onset ± SE.

Because the earliest onset of SD and its higher rate of occurrence in the supragranular layers may be simply due to the fact that KCl is applied at the cortical surface, we also examined propagation of SDs evoked by deep intracortical KCl injection into the infragranular layers (Figures 7A,B). In these experiments, recordings were performed from BCx and KCl was locally injected 3–5 mm caudally at a depth of 1400–1500 μm. The resulting SD evoked by this deep KCl application, was indistinguishable from the epipially-induced SD with the earliest SD onset in the supragranular layers (onset depth at 330 ± 60 μm; *n* = 5) and downstream SD propagation to the granular and infragranular layers (Figures 7C,D). It is noteworthy that similar supragranular SD initiation was also described during a response to the local application of KCl to the infragranular layers in cortical slices *in vitro* (Kaufmann et al., 2017).

## Discussion

Thus, the results of the present study show that silicone probes are suitable for DC-coupled recordings of slow brain activity after compensation of the voltage offset. Exploration of the high-potassium and TBI induced SD in the cortical columns of the visual and barrel cortices using linear multichannel silicone probes revealed preferential SD propagation through the superficial cortical layers. This «tropism» of SD to the superficial layers was evidenced by the earliest onset of SD in the superficial layers 2, 3 and 4 during remote epipial and deep application of KCl, and during SD evoked by TBI (pinprick and dura incision). Also, recurrent SDs during continuous remote application of KCl were mainly restricted to the superficial layers. These results are in keeping with Leão’s findings that SD is more easily induced and propagates faster in the superficial cortical layers (Leão, 1944, 1947), and with a higher frequency of recurrent SDs in the superficial cortical layers (Kaufmann et al., 2017; but also see Richter and Lehmenkühler, 1993). Our findings are also compatible with the results obtained using brain slices, where electrical SD and associated optical intrinsic signal transients preferentially propagate through the superficial layers (Basarsky et al., 1998; Joshi and Andrew, 2001; Kaufmann et al., 2017). We also observed a kind of «barrier» for vertical SD propagation from the superficial to deep cortical layers at a cortical depth of between 800 μm and 1200 μm during continuous KCl application as reported previously (Richter and Lehmenkühler, 1993); however, we didn’t observe SDs restricted to the infragranular layers in the present study.

Vertical propagation of SD in the visual and barrel cortices was characterized by a rapid recruitment of the superficial layers that likely reflects the horizontally moving SD front, followed by slower downward propagation of SD to the granular and infragranular layers at a speed of about 4–6 mm/min. CSD analysis revealed the main sink associated with the rapid initial depolarization, which moved top-down along with the propagation of the LFP shift. Interestingly, the duration of this sink was much shorter than the duration of the negative LFP shift. This dissociation between the sinks and voltage shifts during the time course of SD may be due to an increase in the tissue resistance during SD and error in the estimation of the sink magnitude (Makarova et al., 2008). Overall the depth-profile of SD in the neocortex shared similar features with SD in the hippocampus (Wadman et al., 1992; Herreras and Somjen, 1993a; Herreras et al., 1994; Makarova et al., 2008), although there were also some differences probably due to a difference in cytoarchitectonic organization of the one-layer hippocampus and six-layer neocortex. Although in the neocortex, neurons are distributed through the cortical depth, they have basal-apical organization which mirrors the morphology of the hippocampal pyramidal cells and creates gradients with a higher density of apical dendrites at the superficial layers and basal dendrites in the deep layers. In both structures, onset of SD is characterized by rapid and almost simultaneous LFP shift and an initial frontal sink first in the apical regions (SR and SLM in hippocampus (Makarova et al., 2008) and L2/3 in neocortex (Figure 6C). The next prominent sinks are observed at the level of basal and apical dendrites, and most robustly at the SD offset (Figure 6C). Partially propagating SDs which were restricted to the superficial layers (during the late phase of continuous remote KCl application) correlated particularly well with the SD in hippocampus (Figure 6C, right traces). However, unlike in the hippocampus, SD propagated slowly at a speed characteristic of horizontal SD propagation, during vertical propagation to the deep layers. Also, separation between the apical and basal sinks in neocortex was not as evident as in the hippocampus (Makarova et al., 2008) probably because of diffuse distribution of the pyramidal cell soma in neocortex.

Thus, DC-coupled recordings with silicone probes after offset compensation can be successfully used to explore the spatial—temporal dynamics of SD. Linear silicone probe recordings from the cortical columns in the barrel and visual cortices enabled us to characterize the propagation of SD across layers of the cortical column, and show that KCl and TBI induced SD propagates preferentially through the supragranular layers before invading the entire cortical column and that SD displays prominent layer-specific features. We propose that in future studies, DC-coupled recordings using silicone probes could be useful for spatial-temporal exploration of physiological and pathophysiological slow brain activities including SD in migraine, TBI, brain ischemia and epilepsy.

## Author Contributions

RK conceived the project and wrote the article. AN, NL, GB, KC, DV, JL and AZ performed the experiments. AN analyzed the data and prepared figures.

## Conflict of Interest Statement

The authors declare that the research was conducted in the absence of any commercial or financial relationships that could be construed as a potential conflict of interest.
